# The Heterozygous p.A684V Variant in the *WFS1* Gene Is a Mutational Hotspot Causing a Severe Hearing Loss Phenotype

**DOI:** 10.3390/genes16010057

**Published:** 2025-01-06

**Authors:** Shintaro Otsuka, Chihiro Morimoto, Shin-ya Nishio, Shinya Morita, Daisuke Kikuchi, Masahiro Takahashi, Kozo Kumakawa, Yasuhiro Arai, Hajime Sano, Hidekane Yoshimura, Norio Yamamoto, Shunsuke Kondo, Mari Hasegawa, Tomo Nishi, Tadashi Kitahara, Shin-ichi Usami

**Affiliations:** 1 Department of Otolaryngology-Head and Neck Surgery, Nara Medical University, Kashihara 634-8522, Japan; mori-chi@naramed-u.ac.jp (C.M.); tkitahara@naramed-u.ac.jp (T.K.); 2 Department of Hearing Implant Sciences, Shinshu University School of Medicine, Matsumoto 390-8621, Japan; nishio@shinshu-u.ac.jp; 3 Department of Otolaryngology-Head and Neck Surgery, Hokkaido University, Sapporo 060-8648, Japan; shinyamorita@yahoo.co.jp; 4 Department of Otolaryngology, Fukushima Medical University, Fukushima 960-1295, Japan; kikusuke@fmu.ac.jp; 5 Department of Otorhinolaryngology, International University of Health and Welfare Mita Hospital, Tokyo 108-8329, Japan; masa12_1@iuhw.ac.jp; 6 Department of Otolaryngology, Akasaka Toranomon Clinic, Tokyo 107-0052, Japan; kozo3000@gmail.com; 7 Department of Otorhinolaryngology, Head and Neck Surgery, Yokohama City University, Yokohama 236-0004, Japan; arachan19775245@yahoo.co.jp; 8 Department of Otorhinolaryngology and Head & Neck Surgery, Kitasao University, Sagamihara 252-0375, Japan; sanohj@med.kitasato-u.ac.jp; 9 Department of Otorhinolaryngology-Head and Neck Surgery, Shinshu University School of Medicine, Matsumoto 390-8621, Japan; yoshimura@shinshu-u.ac.jp; 10 Department of Otolaryngology, Kobe City Medical Center General Hospital, Kobe 650-0047, Japan; yamamoto@ent.kuhp.kyoto-u.ac.jp; 11 Department of Otorhinolaryngology, Head and Neck Surgery, University of the Ryukyus, Okinawa 903-0215, Japan; kouhouiinn@yahoo.co.jp; 12 Department of Pediatrics, Nara Medical University, Kashihara 634-8522, Japan; mari-hase@naramed-u.ac.jp; 13 Department of Ophthalmology, Nara Medical University, Kashihara 634-8522, Japan; tomon@naramed-u.ac.jp

**Keywords:** *WFS1*, Wolfram-like syndrome, non-syndromic hearing loss, DFNA6/14/38, genotype–phenotype correlation, optic atrophy, diabetes mellitus, cochlear implant, hearing aid

## Abstract

Background/Objectives: A heterozygous mutation in the *WFS1* gene is responsible for autosomal dominant non-syndromic hearing loss (DFNA6/14/38) and Wolfram-like syndrome, which is characterized by bilateral sensorineural hearing loss with optic atrophy and/or diabetes mellitus. However, detailed clinical features for the patients with the heterozygous p.A684V variant remain unknown. Methods: We report the clinical details of 14 cases with a heterozygous p.A684V variant in the *WFS1* gene identified from target resequencing analysis of 63 previously reported deafness genes by next-generation sequencing of 15,684 hearing loss patients (mean age 27.5 ± 23.1 years old, 6574 male, 8612 female and 498 for whom information was unavailable). Results: Among the 14 patients from 13 families with the p.A684V variant, nine were sporadic cases. In addition, we confirmed de novo occurrence of this variant in seven families. This result strongly supports the notion that this variant was located on a mutational hotspot. When comparing previously reported cases of autosomal dominant *WFS1* gene-associated hearing loss, most of the patients in this study showed severe-to-profound bilateral sensorineural hearing loss (genotype–phenotype correlation). Two patients had optic atrophy, while the others did not have any other complications. Conclusions: The identified heterozygous p.A684V variant appears to be a hotspot mutation and likely to cause severe-to-profound hearing loss in early childhood. Cochlear implantation is considered favorable in cases of hearing impairment due to this variant.

## 1. Introduction

The *WFS1* gene is located on the chromosome 4p16.1, which encodes a transmembrane protein, called wolframin, consisting of 890 amino acids [1]. This gene plays a crucial role in membrane trafficking, protein processing, and the regulation of calcium homeostasis in the endoplasmic reticulum [1]. *WFS1* is expressed in various types of cells in the cochlea and vestibule from the early stage of development and plays an important role in hearing function by maintaining K^+^ and/or Ca^2+^ ion homeostasis in the inner ear [2]. The *WFS1* gene was originally identified as the cause of Wolfram syndrome (MIM # 222300), which is an autosomal recessive disorder characterized by various symptoms including juvenile onset diabetes mellitus, optic atrophy, central diabetes insipidus, psychiatric disease, and hearing impairment [3]. This gene is also responsible for autosomal dominant non-syndromic hearing loss (DFNA6/14/38, MIM # 600965) [4,5] and Wolfram-like syndrome (MIM # 614296), which is characterized by hearing loss with complications of optic atrophy and diabetes mellitus [6,7,8,9]. Although wolframin is equally expressed in both the basal and apical turns of the cochlea, hearing loss manifests differently under various conditions. In DFNA6/14/38 cases, hearing loss primarily affects low frequencies and progresses slowly without reaching a severe-to-profound range [2,4,10,11,12]. It is often associated with tinnitus, but speech perception typically remains good. In contrast, in Wolfram syndrome, hearing loss predominantly impacts high frequencies [12].

The *WFS1*: NM_006005.3: c.2051C>T: p.A684V variant was first identified from a patient with Wolfram syndrome in Italy [13], and was reported in various countries including, the United States [9], Denmark [14], the United Kingdom [15], Japan [10], the Philippines [16], China [17,18], Germany [19], and France [20]. However, detailed clinical phenotypes, including the hearing threshold, progression of hearing loss, optic atrophy, and diabetes mellitus, for patients with the heterozygous p.A684V variant remain unclear. In this study, we reported the detailed clinical information of 14 patients with the heterozygous p.A684V variant identified from 13 independent Japanese families with hearing loss with/without other complications.

## 2. Materials and Methods

### 2.1. Subjects

A total of 15,684 patients with hearing loss were enrolled in this study, and genetic analysis was performed in the Department of Hearing Implant Sciences, Shinshu University School of Medicine. Among the 15,684 patients, 13 probands and one relative were found to carry the heterozygous *WFS1*: NM_006005.3: c.2051C>T: p.A684V variant. Each patient visited one of 10 different medical institutions located across the northern to southern island of Japan (Hokkaido University, Fukushima Medical University, Yokohama City University, International University of Health and Welfare Mita Hospital, Kamio Memorial Hospital, Kitasato University, Shinshu University, Kobe City Medical Center General Hospital, Nara Medical University, and the University of the Ryukyus). The detailed clinical data were collected from medical records. This study was approved by the Shinshu University Ethical Committee (Approval number: No. 387—14 September 2012, No. 576—2 May 2017 and No. 718—7 March 2022), the respective ethical committees of the other participating institutions, and was conducted in accordance with the Declaration of Helsinki. Written informed consent was obtained from all patients (or from their next of kin, caretaker, or guardian in the case of minors or children). We also provided genetic counseling for the parents in the case of minors or children.

### 2.2. Genetic Analysis

To identify the genetic cause of hearing loss, next-generation sequencing analysis was performed. Peripheral blood samples were obtained from probands and their family members, and DNA was extracted using a DNeasy Blood and Tissue Kit (QIAGEN, Hulsterweg, The Netherlands). Next-generation sequencing (NGS) analysis for 63 target deafness genes was performed for all patients. The detailed protocol was described elsewhere [21]. In brief, the amplicon libraries were prepared using an Ion AmpliSeq Custom Panel and Ion AmpliSeq library kit version 2.0 (ThermoFisher Scientific, Waltham, MA, USA). Sequencing was performed using an Ion PGM, Ion Proton, or IonS5 sequencer (ThermoFisher Scientific, Waltham, MA, USA), and the sequence data were mapped against the human genome sequence (build GRCh37/hg19).

The protein-affecting variants (including the missense, nonsense, insertion/deletion, and splicing variants) with an allele frequency of less than 1% of the Genome Aggregation Database ver. 4.1 [22], the 54,000 Japanese genome variation database (ToMMo 54KJPN) [23], and the 333 in-house Japanese normal hearing controls were selected. The annotation for each variant was analyzed by ANNOVAR software version 2020-06-08 [24]. The remaining candidate variants were confirmed by direct sequencing. Segregation analysis for family members was also performed by direct sequencing.

The pathogenicity of the identified variants was evaluated using the American College of Medical Genetics (ACMG) standards and guidelines [25] with the ClinGen hearing loss clinical domain working group’s expert specification [26]. Based on these guidelines and recommendations, *WFS1*: NM_006005.3: c.2051C>T: p.A684V was categorized as a “Pathogenic variant” (PS2_VeryStrong, PM5_Strong, PP1_Strong, PS4_Moderate, PM2, and PP3).

### 2.3. Clinical Evaluation

The hearing thresholds were evaluated using pure-tone audiometry or age-appropriate audiometric methods including auditory brainstem response (ABR), auditory steady state response (ASSR), and conditioned orientation response audiometry (COR). The severity of hearing loss was categorized based on the pure-tone average (PTA: average of hearing thresholds for 500Hz, 1000Hz, 2000Hz, and 4000Hz). Normal hearing was defined as below 25 dB HL. Mild hearing loss was defined as PTA: >25 dB and ≤40 dB HL, moderate hearing loss as >40 dB and ≤70 dB HL, severe hearing loss as >70 dB and ≤90 dB HL, and profound hearing loss was defined as >90 dB HL. Information regarding the intervention for hearing loss, including hearing aids or cochlear implants, was also obtained. We also collected monosyllable perception scores with hearing aid or cochlear implants where relevant. The audiometric configurations were categorized into low-frequency, mid-frequency (U-shaped), high-frequency, flat type, and deaf, as reported previously [27]. The presence of optic atrophy and diabetes mellitus was evaluated during ophthalmologic or metabolic medical follow-ups, respectively.

## 3. Results

### 3.1. Pedigrees, Audiograms, and Clinical Characteristics of Patients with the p.A684V Variant

**Figure 1:**
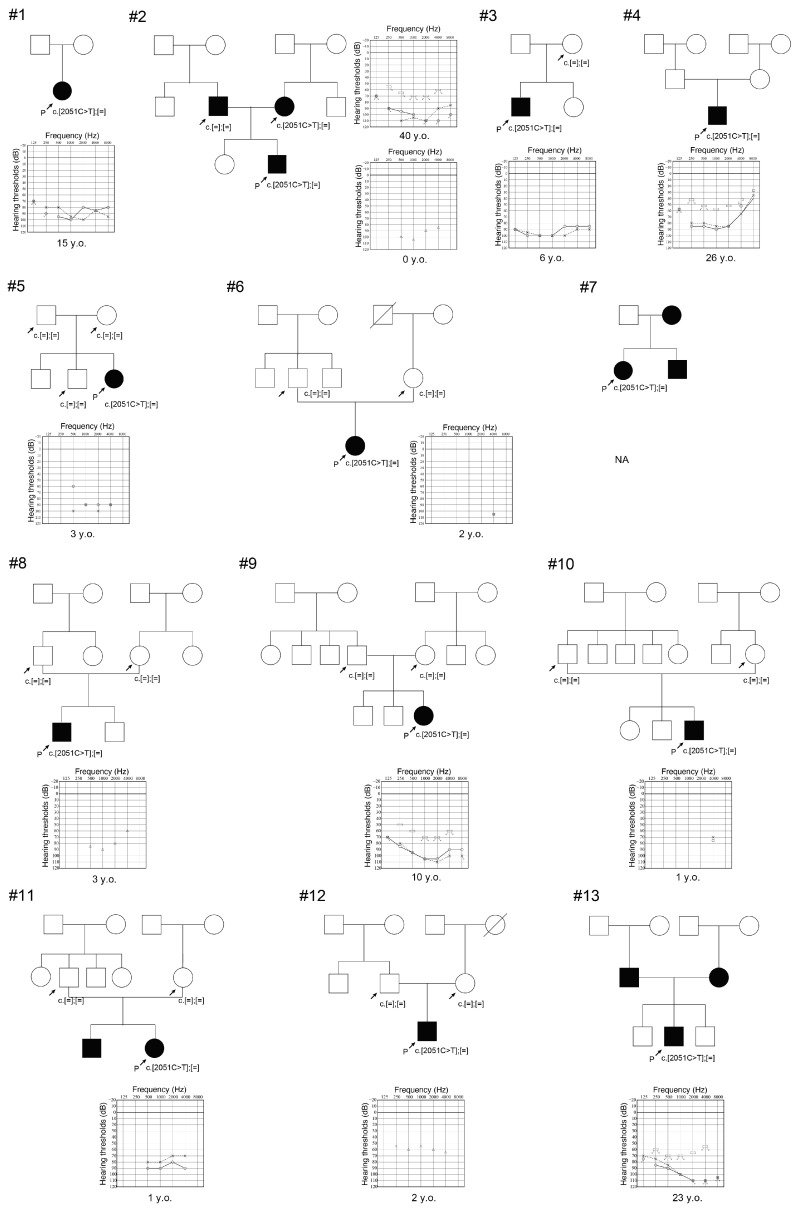
Pedigrees, audiograms, and genetic testing results for patients with the *WFS1* p.A684V variant. Filled symbols indicate affected individuals. Arrows indicate proband and relatives who received genetic analysis. Audiograms indicate hearing threshold for each affected individual with age at which hearing testing was performed.

The pedigrees and audiograms of the 14 patients (seven males, seven females) from 13 families with the *WFS1*: NM_006005.3: c.2051C>T: p.A684V variant are shown in Figure 1. The mean age of the patients was 10.1 ± 11.9 years old. Among the 13 families with the p.A684V variant, nine cases were sporadic cases without any affected relatives. In the results of the genetic analysis of family members, neither of the parents in seven families were found to have this variant, confirming de novo occurrence.

**Figure 2:**
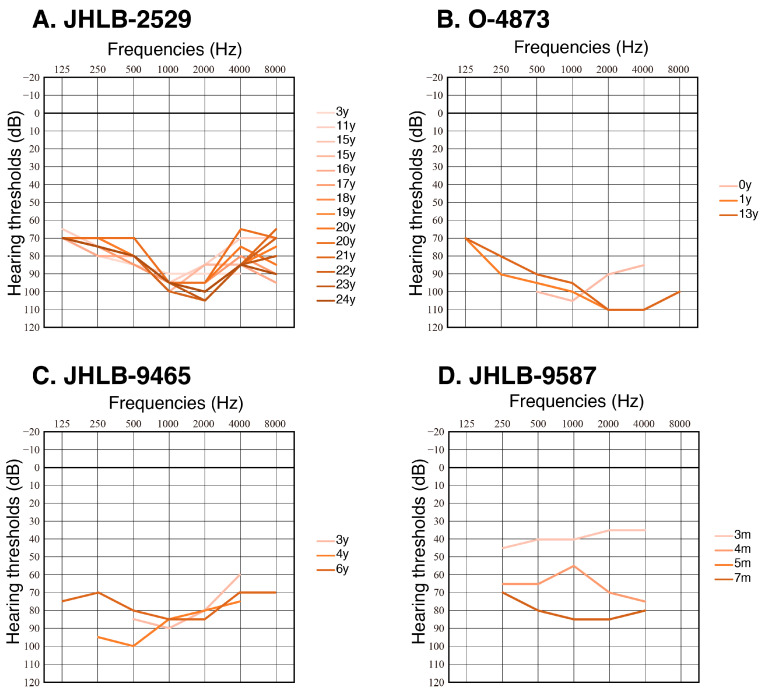
Serial audiograms of four individuals with the *WFS1* p.A684 variant. Lighter colors indicate hearing thresholds at younger ages, and darker colors indicate those at older ages. Vertical axis indicates hearing threshold and horizontal axis indicates frequency.

Clinical findings of the patients are summarized in Table 1. As shown in Table 1, all patients became aware of their hearing loss before two years old, and most of the patients were diagnosed with congenital onset hearing loss. Most of the cases showed severe-to-profound hearing loss and only one patient showed moderate hearing loss. Five out of 13 patients had progressive hearing loss. No one complained of vestibular symptoms. Two out of 11 patients had optic atrophy, but none had diabetes mellitus at the time of genetic testing. Four cases received bilateral cochlear implantation, and seven cases were wearing hearing aids bilaterally. The results of the serial audiometric examinations for four patients (JHLB-2529, O-4873, JHLB-9465, and JHLB-8248) are shown in Figure 2. Detailed clinical information for each family is described below.

### 3.2. Family #1: JHLB-2529 (Female)

JHLB-2529 was a sporadic case with bilateral congenital hearing loss. Her serial audiograms are shown in Figure 2A. She had flat type severe hearing loss from birth, and her hearing levels have been almost stable. She underwent genetic testing at the age of 15, and the heterozygous p.A684V variant in the *WFS1* gene was identified. Her average hearing levels for the left and right ears with hearing aids were 37.5 dB HL and 48.8 dB HL, respectively. She did not have any complications at the time of genetic testing.

### 3.3. Family #2: O-4873 (Male) and O-4875 (O-4873’s Mother)

O-4873, O-4875 (O-4873’s mother), and O-4873’s father all had hearing loss. When the proband was 13 years old, he and his parents underwent genetic testing, and the heterozygous p.A684V variant was detected in the proband and his mother. The proband had congenital bilateral hearing loss. He had flat type severe hearing loss, and his hearing threshold was stable (Figure 2B). His averaged hearing levels for the left and right ears with hearing aids were 32.5 dB HL and 37.5 dB HL, respectively. Neither O-4873 nor O-4875 had any complications.

### 3.4. Family #3: O-2304 (Male)

O-2304 was a sporadic case with congenital onset bilateral hearing loss. He has profound hearing loss and reports that his hearing loss was progressive. He and his mother underwent genetic testing when he was 6 years old, and he was found to carry the heterozygous p.A684V variant but his mother did not. Unfortunately, we could not obtain any information regarding his optic atrophy or hearing intervention.

### 3.5. Family #4: JHLB-2847 (Male)

JHLB-2847 was a sporadic case with congenital onset bilateral low-frequency hearing loss. His hearing threshold was stable. He underwent genetic testing at the age of 26, and the heterozygous p.A684V variant was identified. Unfortunately, we could not obtain any information regarding his optic atrophy or hearing intervention.

### 3.6. Family #5: JHLB-3259 (Female)

JHLB-3259 was a sporadic case without any affected relatives. She had congenital onset bilateral hearing loss. When she was 3 years old, she underwent genetic testing. Genetic testing was also performed for her parents and her brother. A heterozygous p.A684V variant was identified in the proband alone, with her parents and brother not possessing this variant (de novo variant). The averaged hearing level for the bilateral ears with cochlear implants was 33.4 dB HL. She did not have any complications.

### 3.7. Family #6: JHLB-8248 (Female)

JHLB-8248 was a sporadic case with bilateral hearing loss. She passed the Newborn Hearing Screening (NHS); however, auditory brainstem response (ABR) thresholds at the age of 1 year and 11 months were over 100 dB HL bilaterally. She underwent genetic testing at the age of 1 year, and the heterozygous p.A684V variant was identified. Her parents also underwent genetic testing, but they did not have this variant (de novo variant). The averaged hearing levels with bilateral cochlear implants were 30.0 dB HL and 32.5 dB HL, respectively. When she was 6 years old, she was suspected of having a visual problem during a school health checkup; however, her best corrected visual acuity with glasses was good.

### 3.8. Family #7: HL-8085 (Female)

HL-8085 was a familial hearing loss case, and her mother and brother also have hearing loss thought to be autosomal dominant inheritance. The proband underwent genetic testing, and the heterozygous p.A684V variant was identified. Unfortunately, we could not obtain any detailed information for this patient.

### 3.9. Family #8: JHLB-9465 (Male)

JHLB-9465 was a 3-year-old boy with congenital onset low-frequency bilateral hearing loss. His hearing loss was severe and relatively stable (Figure 2C). He and his parents underwent genetic testing, and the heterozygous p.A684V variant was identified only in the proband (de novo variant). The averaged hearing levels with bilateral cochlear implants were 42.5 dB HL and 45 dB HL, respectively. He did not have any complications at the time of genetic testing.

### 3.10. Family #9: JHLB-9649 (Female)

JHLB-9649 was a sporadic case with congenital onset hearing loss. Her hearing loss was profound. When she was 10 years old, she and her parents underwent genetic testing, and the heterozygous p.A684V variant was identified only in the proband (de novo variant). The averaged hearing levels with bilateral cochlear implants were 27.5 dB HL and 22.5 dB HL, respectively. At the time of genetic testing, she did not have any complications, but optic atrophy was identified after genetic analysis.

### 3.11. Family #10: JHLB-9587 (Male)

JHLB-9587 was a 1-year-old boy with bilateral hearing loss. He was a sporadic case without any affected relatives. The ABR thresholds for the left and the right ears were 30 dB HL and 40 dB HL, respectively, at the age of 1 month. However, his hearing deteriorated rapidly to 75 dB HL and 70 dB HL, respectively, at the age of 4 months. The hearing thresholds measured with COR also gradually increased (Figure 2D). When he was 1 year old, he and his parents underwent genetic testing, and the heterozygous p.A684V variant was identified only in the proband (de novo variant). The averaged hearing levels with bilateral cochlear implants have improved to 22.5 dB HL and 27.5 dB HL, respectively. He did not have any complications at the time of genetic testing.

### 3.12. Family #11: JHLB-12110 (Female)

JHLB-12110 was a patient with an autosomal recessive-like family history and her brother also had hearing loss. Her hearing loss was congenital onset profound hearing loss. She and her parents underwent genetic testing when she was aged one, and the heterozygous p.A684V variant was identified only in the proband (de novo variant). Unfortunately, we could not obtain her brother’s DNA sample or hearing threshold information, so the etiology of her brother’s hearing loss remains unclear. Her averaged hearing level with hearing aids was 47.5 dB HL. She did not have any complications.

### 3.13. Family #12: JHLB-12543 (Male)

JHLB-12543 was a congenital onset bilateral hearing loss patient without any family history. His hearing loss was moderate and stable. When he was 2 years old, he and his parents underwent genetic testing, and the heterozygous p.A684V variant was identified only in the proband (de novo variant). His averaged hearing level with hearing aids was 35 dB HL. He did not have any complications.

### 3.14. Family #13: JHLB-13616 (Male)

JHLB-13616 was a 23-year-old male with congenital onset bilateral high-frequency gently sloping hearing loss. Both of his parents had hearing loss, but the inheritance pattern from his family history was unknown. He underwent genetic testing at the age of 23 years, and the heterozygous p.A684V variant was identified. He had visual symptoms from 17 years old that were diagnosed as optic atrophy.

## 4. Discussion

Here, we reported the findings for 14 hearing loss patients from 13 independent Japanese families with the heterozygous p.A684V variant in the *WFS1* gene. In nine out of the 13 families, the probands were sporadic cases without any affected family members, and in seven families, we confirmed this variant occurred as a de novo variant (neither of the parents carried this variant). This high prevalence of de novo occurrence for this variant strongly suggests that the p.A684V variant is a hotspot mutation. This hypothesis is also supported by the fact that this variant was identified in hearing loss patients from many different ethnic backgrounds.

**Figure 3:**
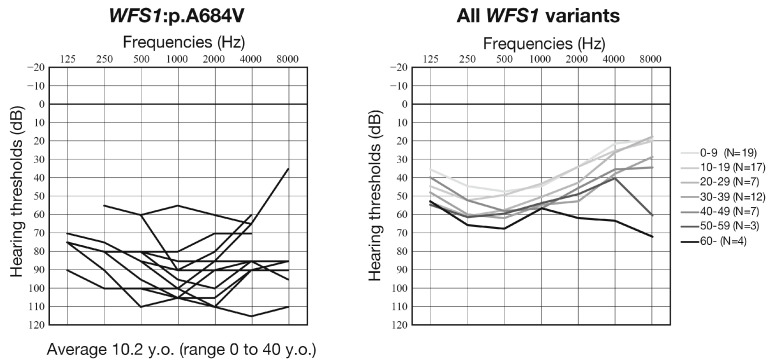
Overlapping audiograms of patients with the *WFS1* p.A684 variant identified in this study and averaged hearing thresholds in each age group of all autosomal dominant *WFS1*-associated hearing loss patients in our previous report [28]. Vertical axis indicates hearing threshold, and horizontal axis indicates frequency.

With regard to the clinical details of the patients with this variant, we clarified that most of the patients had severe-to-profound sensorineural hearing loss (Figure 1, Table 1). In our previous study, we performed massively parallel DNA sequencing analysis for 10,047 independent Japanese patients with hearing loss and identified 94 cases with autosomal dominant *WFS1* gene-associated hearing loss [28]. Interestingly, the average audiogram of these cases showed mild-to-moderate low-frequency hearing loss (Figure 3), which is a well-known clinical characteristic of autosomal dominant *WFS1* gene-associated hearing loss (DFNA6/14/38) [2,4,10]. This result clearly indicates that the p.A684V variant is correlated with a more severe hearing loss phenotype than other forms of autosomal dominant *WFS1* gene-associated hearing loss (genotype–phenotype correlation). It is worth noting that one of our patients (Family #10: JHLB-9587) showed rapid hearing deterioration between 3 and 5 months of age (Figure 2D), and this may indicate the progressive nature of hearing loss associated with this gene variant. Therefore, continuous follow-ups and auditory assessments will be important for mild-to-moderate hearing loss patients with this gene variant.

As for the clinical intervention for hearing loss, five patients wore bilateral hearing aids, and their hearing levels were improved to 40.9 dB in average (ranging from 32.5 dB to 48.8 dB). The other four patients received bilateral cochlear implantation, and their hearing levels were improved to 28.7 dB on average (ranging from 22.5 dB to 33.4 dB). Our cases indicated that hearing aids are effective to some extent; however, insufficient amplification was observed in some patients. Based on the severe-to-profound hearing loss phenotype associated with this variant, cochlear implantation is considered the most favorable treatment for patients with this variant.

With regard to the optic atrophy and diabetes mellitus that characterize Wolfram-like syndrome, only two patients had optic atrophy, while none of the others had any ophthalmic complications in this study. In previous studies, Rendtorff et al. reported seven patients with the heterozygous p.A684V variant from six independent European and American families [14]. They reported that six patients were autosomal dominant cases, and one was a sporadic case. They showed congenital or progressive sensorineural hearing loss occurring in early childhood, and the types of hearing loss were flat type or mid-frequency. All seven patients had optic atrophy, and patients from two families also had psychiatric symptoms (including hallucinations and depression). In their report, optic atrophy generally appeared as late onset in their childhood or teenage years, while some cases were diagnosed with optic atrophy in their 30s or 40s. A recent systematic review of Wolfram-like syndrome also supports the notion that the hearing loss associated with Wolfram-like syndrome occurs within the first decade; however, optic atrophy commonly occurs in the second decade [29]. One of the plausible reasons for the small number of patients with optic atrophy in this study may be the relatively younger patient age (average 10.2 years). However, some patients over 20 years of age did not have optic atrophy, so other factors including other genetic variants may affect the onset of optic atrophy. Nevertheless, optic atrophy associated with the *WFS1* gene p.A684V variant may be late onset, and ophthalmologic follow-up is necessary in the consideration of the possibility of late onset optic atrophy.

No patients with diabetes mellitus were observed in this study or in the previous reports of p.A684V heterozygous variant cases. However, other heterozygous variants in the *WFS1* gene are suspected to have an association with diabetes mellitus. Eiberg et al. reported four cases in one family with a p.E864K heterozygous variant in the *WFS1* gene. One of them had progressive sensorineural hearing loss with optic atrophy, while the other three cases had diabetes mellitus or impaired glucose tolerance [6]. Domènech et al. reported hearing loss patients with heterozygous p.D729N, p.L757I, and p.V871M variants in the *WFS1* gene. Bilateral hearing loss patients with p.D729N and p.L757I variants from two independent families also had type 2 diabetes mellitus. One patient with the p.V871M variant also had bilateral sensorineural hearing loss and type 2 diabetes mellitus. However, three cases with the p.V871M variant from the other family did not have diabetes mellitus [8]. Further validation is required to clarify the relationship between diabetes mellitus and the variants, as various phenotypes were observed, even among patients with the same variant. Although there are no reported cases with diabetes mellitus among patients with the p.A684V variant, long-term monitoring of blood glucose levels may be necessary to avoid overlooking symptoms.

As a limitation to this study, most of the patients in this study were under 10 years old, so further prospective studies will be needed to more accurately clarify the frequencies of optic atrophy and diabetes.

## 5. Conclusions

In conclusion, the heterozygous p.A684V variant in the *WFS1* gene causes congenital or early childhood onset severe-to-profound hearing loss. Cochlear implantation is considered a good treatment option for patients with this mutation. In addition, the p.A684V variant in seven of 10 sporadic cases was de novo and indicates a mutational hotspot in this gene. Moreover, long-term ophthalmologic and metabolic medical follow-up is also strongly recommended due to the possibility of late onset optic atrophy and diabetes mellitus.

## Figures and Tables

**Table 1:** Clinical characteristics of patients with the *WFS1* p.A684V variant.

Family Number	ID	Relationship	Hereditary	Onset	Age	Gender	Severity of HL	Type of HL	Progression	Vestibular Symptoms	Optic Atrophy	DM	Intervention	Hearing Threshold with HA/CI (R)	Hearing Threshold with HA/CI (L)	Monosyllable Perception Score with HA/CI (R)	Monosyllable Perception Score with HA/CI (L)
1	JHLB-2529	Proband	Sporadic	0	15	F	Severe	Flat	N	N	N	N	Bilateal HA	48.8 dB	37.5 dB	50%	65%
2	O-4873	Proband	Unknown	0	0	M	Severe	Flat	N	N	N	N	Bilateal HA	37.5 dB	32.5 dB	NA	NA
	O-4875	Mother		2	40	F	Severe	Flat	N	N	N	N	Bilateal HA	NA	NA	NA	NA
3	O-2304	Proband	Sporadic	0	6	M	Profound	Flat	Y	N	NA	N	NA	NA	NA	NA	NA
4	JHLB-2847	Proband	Sporadic	0	26	M	Profound	Lo freq	N	N	NA	N	NA	NA	NA	NA	NA
5	JHLB-3259	Proband	Sporadic (de novo)	0	3	F	Severe	Flat	N	N	N	N	Bilateal CI	33.4 dB	33.4 dB	92%	92%
6	JHLB-8248	Proband	Sporadic (de novo)	1	2	F	Profound	Flat	Y	N	N	N	Bilateal CI	32.5 dB	30 dB	50%	50%
7	HL8085	Proband	AD	NA	NA	F	NA	NA	NA	NA	NA	NA	NA	NA	NA	NA	NA
8	JHLB-9465	Proband	Sporadic (de novo)	1	3	M	Severe	Lo freq	Y	N	N	N	Bilateal HA	45 dB	42.5 dB	50%	70%
9	JHLB-9649	Proband	Sporadic (de novo)	0	10	F	Profound	Flat	Y	N	Y	N	Bilateal CI	22.5 dB	27.5 dB	NA	NA
10	JHLB-9587	Proband	Sporadic (de novo)	0	1	M	Severe	Flat	Y	N	N	N	Bilateal CI	27.5dB	22.5dB	NA	NA
11	JHLB-12110	Proband	AR (de novo)	0	1	F	Severe	Flat	N	N	N	N	Bilateal HA	47.5 dB	47.5 dB	NA	NA
12	JHLB-12543	Proband	Sporadic (de novo)	0	2	M	Moderate	Flat	N	N	N	N	Bilateal HA	35 dB	35 dB	NA	NA
13	JHLB-13616	Proband	Unknown	0	23	M	Profound	HF gentle	N	N	Y	N	Bilateral HA	NA	NA	NA	NA

HL: hearing loss; DM: diabetes mellitus; AD: autosomal dominant; AR: autosomal recessive; M: male; F: female; Y: yes; N: no; NA: not available; CI: cochlear implant; HA: hearing aid; R: right ear; L: left ear.

## Data Availability

The datasets used during the current study are available from the corresponding author on reasonable request.
